# Early detection of hypervascularization in hepatocellular carcinoma (≤2 cm) on hepatic arterial phase with virtual monochromatic imaging: Comparison with low-tube voltage CT

**DOI:** 10.1097/MD.0000000000034774

**Published:** 2023-09-29

**Authors:** Haruomi Yamaguchi, Tomoaki Ichikawa, Hiroyuki Morisaka, Hiroyuki Akai, Keisuke Izuka, Takashi Ueno, Osamu Abe, Yoshito Tsushima

**Affiliations:** a Department of Radiology, Institute of Medical Science, University of Tokyo, Tokyo, Japan; b Diagnostic Radiology and Nuclear Medicine Department, Gunma University Graduate School of Medicine, Gunma, Japan; c Department of Radiology, University of Yamanashi, Yamanashi, Japan; d Department of Gastroenterology, Isesazaki Municipal Hospital, Gunma, Japan; e Department of Radiology, Graduate School of Medicine, University of Tokyo, Tokyo, Japan.

**Keywords:** dual-energy CT, hepatocellular carcinoma, iodine, low-tube voltage CT, virtual monochromatic imaging

## Abstract

This study aims to assess the diagnostic value of virtual monochromatic image (VMI) at low keV energy for early detection of small hepatocellular carcinoma (HCC) in hepatic arterial phase compared with low-tube voltage (80 kVp) CT generated from dual-energy CT (DE-CT). A total of 107 patients with 114 hypervascular HCCs (≤2 cm) underwent DE-CT, 140 kVp, blended 120 kVp, and 80 kVp images were generated, as well as 40 and 50 keV. CT numbers of HCCs and the standard deviation as image noise on psoas muscle were measured. The contrast-to-noise ratios (CNR) of HCC were compared among all techniques. Overall image quality and sensitivity for detecting HCC hypervascularity were qualitatively assessed by three readers. The mean CT numbers, CNR, and image noise were highest at 40 keV followed by 50 keV, 80 kVp, blended 120 kVp, and 140 kVp. Significant differences were found in all evaluating endpoints except for mean image noise of 50 keV and 80 kVp. Image quality of 40 keV was the lowest, but still it was considered acceptable for diagnostic purposes. The mean sensitivity for detecting lesion hypervascularity with 40 keV (92%) and 50 keV (84%) was higher than those with 80 kVp (56%). Low keV energy images were superior to 80 kVp in detecting hypervascularization of early HCC.

## 1. Introduction

The distinct sequential stages in hepatocellular carcinoma (HCC) development have a consensus.^[1]^ HCC develop on a step-by-step basis according to hepatocarcinogenesis has been deeply built, that is HCC proceed in stages from hypovascular nodules to hypervascular, advanced HCC. The appearance of hypervascularization within hypovascular early HCC was reported when the tumor exceeds 15 to 20 mm in size.^[2,3]^ Generally, HCC is treated after confirming hypervascularity within the nodules (advanced HCC).^[4–6]^ Therefore, detection of hypervascularization at the earliest stage is the key to improving therapeutic outcomes of HCC.

Hepatic arterial phase (HAP) images with multiphasic contrast enhancement computed tomography (CE-CT) are widely used for hypervascular HCC detection.^[1–3,7]^ However, HAP images with CE-CT may have poor sensitivity for hypervascular HCC detection because early-stage hypervascular HCC may have only a subtle arterial supply.^[8–10]^ Moreover, several reports have shown that the detectability of early HCC enhancement at HAP of gadolinium-ethoxybenzyl-diethylenetriamine pentaacetic acid-enhanced magnetic resonance imaging (EOB-MRI) is superior to CE-CT.^[11,12]^ However, MRI has contraindications such as metallic implants, and also MRI is less accessible considering their higher costs and longer scanning times. Therefore, establishing a more sensitive CT technique is strongly required considering the inadequate diagnostic performance of HAP images with single-energy CT (SE-CT) techniques.

Some studies have shown that single-energy low-tube voltage CE-CT improves hypervascular HCC detection during HAP.^[13]^ Recently, virtual monochromatic imaging (VMI) with dual-energy CE-CT imaging (DE-CT) has been introduced, and VMI at low keV is reported to show a similar contrast enhancement CT numbers of the abdominal organs as with SE-CT imaging with a dramatically reduced iodine dose.^[14–16]^ However, no studies have investigated the use of VMI in the detection of hypervascularization of early HCC.

We hypothesized that VMI at low keV will improve the detectability for small HCC hypervascularity compared with low-tube voltage CT. This study investigated the effectiveness of VMI at low keV (40 and 50 keV) in detecting small HCC (≤20 mm) hypervascularity during HAP in comparison with high (140 kVp), standard (120 kVp), and low-tube voltage (80 kVp) images.

## 2. Material and methods

### 2.1. Patients

This retrospective study reviewed all available DE-CT with VMI reconstruction of the liver at our institute from March 2020 to February 2021. This study was approved by the institutional review board of our hospital. Informed consent was waived because this study used routine CE-CT examination data. Patient records and information were anonymized and deidentified before analysis.

DE-CT was conducted in 963 patients with cirrhosis with suspected HCC based on ultrasound (US) findings and elevated tumor markers. Of these 963 patients, 536 patients were excluded because they did not undergo a combination of EOB-MRI and rotational digital subtraction angiography (DSA) followed by iodized-oil CT imaging or EOB-MRI and the contrast-enhanced US, which were used as the gold standard for confirming the lesions and hypervascularity within the lesions. For the remaining 427 patients, following criteria were used to select small HCC: ≤ 2 cm in size; Hypointensity on hepatocyte phase of EOB-MRI > 6 months ago to confirm the hypovascular to hypervascular process; Exclusion of other focal hepatic lesions (cyst, hemangioma, metastasis, and pseudolesions, such as arterio-portal shunt) by a combination of US and/or routine MRI; and Increase in lesion size (>20% of the initial size) on hepatocyte phase of EOB-MRI. Of these 427 patients, 320 patients were excluded because of above selection criteria. Finally, 107 patients (73 males, 34 females; mean age: 73 years [range: 56–87]; mean body weight: 59 kg [range: 34–68]) with 114 small hypervascular HCCs (range: 5–20 mm; mean: 14 mm; median: 12 mm) were enrolled (97 patients with 1 lesion, 4 with 2 lesions, and 3 with 3 lesions).

The HCC diagnosis clinical flow in our hospital is that physicians pick up patients with cirrhosis based on non-enhanced US and elevated tumor markers. And the patients with suspected HCC undergo DE-CT or EOB-MRI each 3 months. After HCC is detected with DE-CT or EOB-MRI, the patients undergo complement DE-CT or EOB-MRI before treatment. Before radiofrequency ablation, CE-US are performed for patients.

### 2.2. Reference standards of lesions

Of the 114 small HCCs, 5 resected lesions were diagnosed as HCC based on histopathological examination, and the remaining 109 lesions were diagnosed as hypervascular HCC based on the following imaging criteria: Presence of early contrast enhancement on HAP images with EOB-MRI or contrast-enhanced US and/or^[2]^ focal contrast enhancement combination on the rotational DSA images and nodular iodized-oil deposition on the iodized-oil CT images obtained 2 to 4 weeks after the rotational DSA examinations.^[17]^

### 2.3. DE-CT with VMI reconstruction

All multiphasic CE-CT were conducted within 2 months from EOB-MRI, CE-US, or rotational DSA examination. All the CT scans were conducted with a dual-source MDCT scanner (SOMATOM Definition Flash; Siemens Healthcare, Forchheim, Germany) with a maximum tube current of 1600 mA (2 × 800 mA) for single-energy use or a combination of 650 mA at tube A (80 kVp) and 714 mA at tube B (140 kVp) for dual-energy use; rotation speed of 0.5 seconds; pitch factor of 0.6; detector configuration of 0.6 mm × 128-slice detector; and field of view of 400 mm. All patients underwent CE-CT, including unenhanced, HAP, portal venous phase (PVP), and delayed phase (DP) images. The tube current was automatically controlled by an automatic-exposure control software (CARE Dose4D, Siemens Healthcare, Forchheim, Germany). The patients intravenously received body weight-tailored volume (600 mgI/kg) of iomeprol (Iomeron, 350 mgI/mL, Eizai-Bracco, Tokyo, Japan) or iopamidol (Iopamiron, Bayer Yakuhin Ltd, Osaka, Japan) with a power injector (Dual Shot GX, Nemotokyorindo, Tokyo, Japan). The contrast material was administered using a monophasic injection technique during a fixed injection duration of 30 seconds in all patients.^[10]^ The scan timing for HAP, PVP, and DP imaging was 18, 55, and 160 seconds, respectively, after the trigger by the computer-assisted bolus-tracking system with a threshold of 150 HU of the abdominal aorta at the L1 vertebral body level.

For DE-CT, 80 and 140 kVp were utilized as low and high tube voltages, respectively. Transverse HAP images were obtained with 140 kVp, 80 kVp, and blended 120 kVp, of which the latter were considered standard diagnostic HAP images, which were reconstructed with 2 and 5 mm slice thickness, both gapless. Additionally, 2 different VMI obtained at 50 and 40 keV were reconstructed with 2 and 5 mm slice thickness and 2 and 5 mm intervals. VMI energy levels were chosen considering that one of the study purposes was to compare VMI with 80 kVp images; 50 keV was considered to have similar contrast-enhanced effect compared with that of 80 kVp. We utilized the dedicated monoenergetic image reconstruction algorithm technique (Monoenergetic Plus; Siemens Healthcare, Forchheim, Germany) that can significantly increase iodine contrast at low keV without an increased image noise at a radiation dose level similar to SE-CT imaging in an attempt to offset the higher image noise that is possibly seen in VMI obtained from DE-CT data.^[18]^

### 2.4. Image analyses

#### 2.4.1. Quantitative assessment

Two radiologists, with 32 and 9 years of abdominal CT experience, respectively, made the quantitative CT measurements (in HU) for all patients by consensus. The regions of interest (ROIs) were placed on the HCC, the abdominal aorta and the main portal vein at the hepatic hilum level as large as possible on the HAP images with each CT technique with 5mm slice thickness. We placed circular ROI on paraspinal muscles at the level of L1 vertebral body level and calculated the standard deviation to evaluate the image noise with 5 mm slice thickness. Circular ROIs were placed at 3 sites (left lobe and the anterior and posterior segments of the right lobe) at the level of the hepatic hilum with careful exclusion of blood vessels, intrahepatic bile ducts, and artifacts for the normal liver parenchyma. The CT measurements in ROIs were performed twice, and the average CT numbers were calculated for each structure. The contrast-to-noise ratio (CNR) was defined by the following equation: [postcontrast attenuation CT number of the lesion − postcontrast attenuation CT number of the liver parenchyma]/image noise.

#### 2.4.2. Qualitative assessment

All HAP images were independently interpreted in random order by 3 reviewers with 32, 16, and 9 years of abdominal CT experience, respectively, on imaging monitors. Three readers were blinded to the image type, clinical history and histopathological evaluations. During the review, the reviewers could change the image window width/level at their discretion. The reviewers could also reference unenhanced PVP and DP of dynamic CT and all EOB-MRI phases and sequences. After excluding other focal hepatic lesions (cyst, hemangioma, metastasis, and pseudolesions, such as arterio-portal shunt), the reviewers evaluated the appearance of early enhancement within these hypovascular nodules confirmed by EOB-MRI and the presence (or absence) of hypervascular lesions was graded on a 5-point confidence scale (Definitely absent; Probably absent; Equivocal; Probably present; and Definitely present). A lesion assigned to grades 4 or 5 (probably or definitely present) was regarded as positive. A lesion was assigned to grades 1, 2, or 3 and was regarded as negative. In this sensitivity analysis, the slice thickness was 2 mm. Also, the reviewers subjectively rated the overall image quality using a 5-point scale (1 = nondiagnostic, 2 = poor, 3 = fair, 4 = good, and 5 = excellent) for all HAP images obtained with each CT technique. Images that were considered acceptable for clinical diagnostic use were awarded scores of 3 to 5. In this overall image quality analysis, the slice thickness was 5 mm.

### 2.5. Statistical analyses

All statistical analyses were conducted using Statistical Package for the Social Sciences software (version 13.0 for Windows, SPSS Inc., Chicago, Illinois) with a *P* value of < .05 indicating a statistically significant difference. In the present study, both quantitative CT measurements and qualitative image analysis did not show a normal distribution by Shapiro–Wilk test; the Friedman test and post hoc analysis using Dunn multiple comparison test were used for comparison.

The degree of interobserver agreement between 3 reviewers for overall image quality and sensitivity for small HCC hypervascularization was calculated using the Fleiss kappa statistics. Generally, a Fleiss kappa statistic of 0.81 to 1.00 is considered almost perfect agreement; 0.61 to 0.80, substantial agreement; 0.41 to 0.60, moderate agreement; 0.21 to 0.40, fair agreement; 0.01 to 0.20, slight agreement; and < 0, poor agreement.^[19,20]^

## 3. Results

### 3.1. Dose of radiation exposure in DE-CT imaging

The mean CTDI vol (9.86 ± 1.62 mGy) and DLP (200.3 ± 50.1 mGycm) were within the allowable range in all examinations.

### 3.2. Mean image noise, CNR, and mean attenuation values for lesions and each organ

Table 1 summarizes the mean image noise, CNR, and the mean attenuation values of the lesion, liver parenchyma, aorta, and portal vein on HAP images for each technique. Significant differences were found in all categories between each technique (*P* < .001) except for the image noise between the 80 kVp and the VMI at 50 keV (*P *= .818).

**Table 1:** Image noise, CNR, and CT attenuation values for small HCC and each organ on HAP images obtained with five different techniques using DE-CT.

	140 kVp	Blended 120 kVp	80 kVp	VMI at 50 keV	VMI at 40 keV	*P* value*
Image noise (SD [HU])	9.4 ± 1.9	11.4 ± 2.0	13.9 ± 2.5†	14.7 ± 2.6†	19 ± 2.9	<.001
CNR	0.68 ± 1.2	2.6 ± 2.2	3.6 ± 2.9	5.2 ± 3.6	6.2 ± 4.4	<.001
HCC [HU]	80.4 ± 14.0	102.1 ± 20.1	136.7 ± 39	171.2 ± 50.0	230.6 ± 77.1	<.001
Liver [HU]	72.8 ± 9.2	78.4 ± 10.1	88.5 ± 15.9	98.8 ± 21.5	118 ± 34.5	<.001
Aorta [HU]	196.6 ± 30.6	317.4 ± 46.1	495.4 ± 74.4	703.5 ± 105.8	1044.5 ± 156.6	<.001
Portal vein [HU]	94.3 ± 29.9	138.9 ± 51.4	203.5 ± 82	277.1 ± 115.9	396.1 ± 180.5	<.001

Data presented as mean ± standard deviation.

CNR = contrast-to-noise ratio, DE-CT = dual-energy CT imaging, HAP = hepatic arterial phase, HCC = hepatocellular carcinoma, VMI = virtual monochromatic imaging.

* *P* values indicate statistical significance for the difference among the five different HAP images by the Friedman test.

† In the post hoc analysis using Dunn multiple comparisons, all comparisons among the five different CT techniques were statistically significant for all variables except for the difference between the image noise of 80 kVp and VMI at 50 keV (*P* = .818).

### 3.3. Mean values for overall image quality on hap images obtained with 5 different techniques using DE-CT imaging

Table 2 summarizes the mean values for the overall image quality for each technique. The post hoc analysis using the Wilcoxon signed-rank test revealed higher mean values for 140 and 120 kVp than those for 80 kVp and VMI at 50 and 40 keV (*P* < .05). The mean values for 80 kVp and VMI at 50 keV were higher than that for VMI at 40 keV (*P* < .05). The mean value for VMI at 40 keV was the lowest among all techniques, and evaluating the overall image by 40 keV image is not easy.

**Table 2:** Mean values for overall image quality on HAP images obtained with five different techniques using DE-CT.

	140 kVp†	Blended 120 kVp†	80 kVp	VMI at 50 keV	VMI at 40 keV‡	*P* value*
Reader 1	4.82 ± 0.4	4.74 ± 0.5	3.62 ± 0.8	3.62 ± 0.8	3.44 ± 0.7	<.001
Reader 2	4.71 ± 0.5	4.63 ± 0.5	3.64 ± 0.8	3.62 ± 0.8	3.41 ± 0.8	<.001
Reader 3	4.64 ± 0.5	4.56 ± 0.6	3.49 ± 0.8	3.53 ± 0.7	3.34 ± 0.8	<.001

Data presented as mean ± standard deviation.

DE-CT = dual-energy CT imaging, HAP = hepatic arterial phase, VMI = virtual monochromatic imaging.

* *P* values indicate statistical significance for the difference among the five different HAP images by the Friedman test.

† In the post hoc analysis using the Dunn multiple comparison test, the mean values for 140 kVp and the blended 120 kVp were higher than those for 80 kVp and VMI at 50 keV and 40 keV (*P* < .05).

‡ The mean values for VMI at 40 keV were lower than those for the other four techniques (*P *< .05).

### 3.4. Sensitivity for detecting hypervascularity within lesions on HAP images using each technique

Table 3 shows the results of objective hypervascularization detection in lesions for HAP images with each technique.

**Table 3:** Sensitivity for hypervascularization in small HCC on HAP images obtained with five techniques using DE-CT.

	140 kVp	Blended 120 kVp	80 kVp	VMI at 50 keV†	VMI at 40 keV†	*P* value*
Reader 1	16/114 (14)	41/114 (36)	68/114 (60)	100/114 (88)	105/114 (92)	<.001
Reader 2	13/114 (11)	36/114 (32)	63/114 (55)	92/114 (81)	102/114 (89)	<.001
Reader 3	12/114 (10)	38/114 (33)	61/114 (53)	95/114 (83)	106/114 (93)	<.001
Mean	14/114 (12)	38/114 (33)	64/114 (56)	96/114 (84)	104/114 (92)	<.001

The numbers in the parenthesis are percentages.

DE-CT = dual-energy CT imaging, HCC = hepatocellular carcinoma, HAP = hepatic arterial phase, VMI = virtual monochromatic imaging

* *P* values indicate statistical significance for the difference among the five techniques by the Friedman test.

† In the post hoc analysis using the Dunn multiple comparison test, all comparisons among the five techniques were statistically significant for the three readers except for the difference between the sensitivities of VMI at 50 keV and VMI at 40 keV (*P* = .262–0.286).

**Figure 1.**
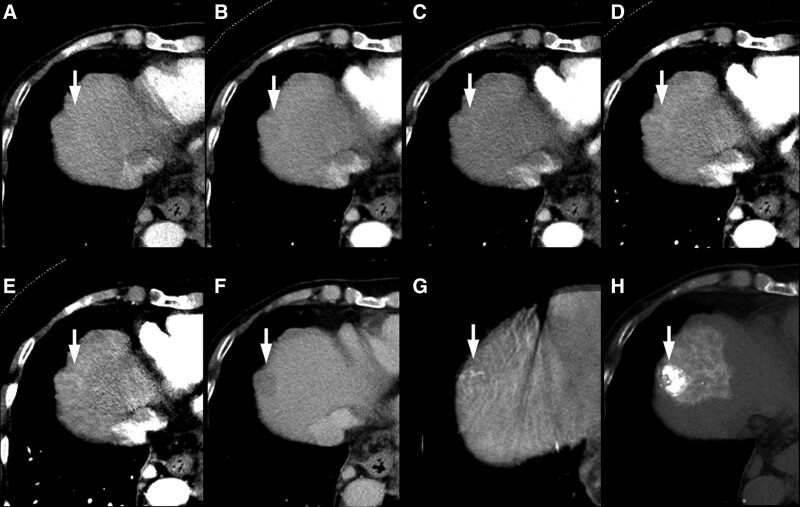
An 84-year-old female patient with slightly hypervascular small HCC. HAP images with (A) 140 kVp, (B) blended 120 kVp, and (C) 80 kVp and VMI at (D) 50 keV and (E) 40 keV, (F) DP SE-CT image, (G) angiographic CT-like image, and (H) post-transcatheter arterial chemoembolization therapy iodized-oil CT image are shown. Sensitivity scores for early HCC enhancement were 1, 1, 3, 4, and 5 in order from (A) to (E), respectively, and the hypervascularity was only detectable with low keV VMI images. DP = delayed phase, HAP = hepatic arterial phase, HCC = hepatocellular carcinoma, SE-CT = single-energy CT, VMI = virtual monochromatic imaging.

**Figure 2.**
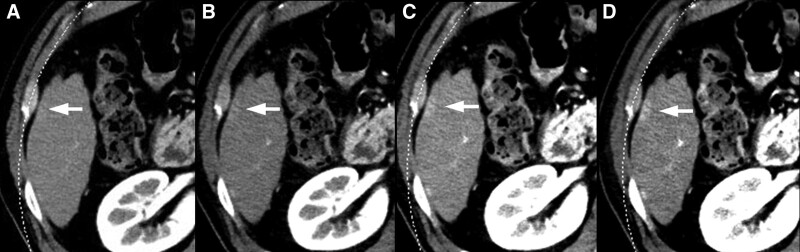
A 75-year-old male patient with slightly hypervascular small HCC. HAP images with (A) blended 120 kVp, (B) 80 kVp, and VMI at (C) 50 keV and (D) 40 keV. Sensitivity scores for early HCC enhancement were 2, 3, 4, and 5 in order from (A) to (D), respectively. HAP = hepatic arterial phase, HCC = hepatocellular carcinoma, VMI = virtual monochromatic imaging.

VMI at 40 keV (102–106/114, 89%–93%) showed the highest sensitivity followed by VMI at 50 keV (92–100/114, 81%–88%), 80 kVp (61–68/114, 53%–60%), blended 120 kVp (36–41/114, 32%–36%), and 140 kVp (12–16/114, 10%–14%) (*P *< .0001) (Figs. 1 and 2). The sensitivities among the 5 techniques were statistically significantly different in the 3 readers, except for the difference between VMI at 50 and 40 keV (*P* = .262–0.286).

### 3.5. Interobserver agreement for image interpretation

The Fleiss Kappa values of the overall image quality and sensitivity for small HCC hypervascularization were 0.80 and 0.61, which are considered substantial agreement (kappa = 0.61–0.80) for interpreting all images types.

## 4. Discussion

Detecting the emergence of small early enhancement within early (hypovascular) HCC is important from the perspective of therapeutic strategy.^[5,6]^ Some studies reported that EOB-MRI was superior to CE-CT in detecting early HCC enhancement.^[12,21]^ However, evaluation of all high-risk patients with HCC with EOB-MRI alone may not be feasible, considering the relative paucity of equipment and lower throughput of EOB-MRI than CE-CT. Moreover, a certain number of HAP images of EOB-MRI were undiagnostic quality because of gadoxetic acid-induced transient dyspnea.^[22–25]^ Improving the detectability for early HCC enhancement with CE-CT may cover these EOB-MRI disadvantages. Therefore, the present study aimed not to investigate the overall diagnostic performance but to assess the sensitivity of HAP images alone in early HCC hypervascularization. Increasing the contrast enhancement has 2 methods, namely, a low-energy image of VMI and a low-tube voltage image. The present study compared these 2 methods in detectability for early small HCC enhancement.

The mean CT numbers for each organ were higher at 40 keV, 50 keV, 80 kVp, blended 120 kVp, and 140 kVp, and the mean imaging noise (standard deviation) for each energy image was higher at 40 keV, 50 keV, 80 kVp, blended 120 kVp, and 140 kVp, in order, in this study. The mean CNR values were higher at 40 keV, 50 keV, 80 kVp, blended 120 kVp, and 140 kVp; thus, increased CT numbers in VMI at low-energy had more impact on CNR improvement than increased image noise. Supporting CNR results, the detection rate was highest in 40 keV (92%), followed by 50 keV (84%), 80 kVp (56%), blended 120 kVp (33%), and 140 kVp (12%): VMI at low-energy images (40 and 50 keV) were more effective than low-tube voltage image (80 kVp) in early HCC enhancement detection.

The mean values for overall image quality revealed higher mean scores of 140 kVp (4.72) and blended 120 kVp (4.64) among 3 readers than those of 80 kVp (3.58), 50 keV (3.59), and 40 keV (3.40). The 5-point scale rated the 3-point as fair; thus, 80 kVp/50 keV/40 keV images were unlikely to have significant image quality degradation that would affect early HCC enhancement detectability. Indeed, the overall image quality of the qualitative assessment revealed that the rate of score 2 in 40 keV images was only 20/342 (6%) with 3 readers and did not limit the early HCC enhancement detection.

Comparing the 80 kVp and VMI at 50 keV, both the mean sensitivity and CNR were superior for VMI at 50 keV, whereas no significant difference was found in the mean image noise between the 2 techniques. VMI may be useful in detecting the early HCC enhancement developing from hypovascular nodules (e.g., early HCC or high-grade dysplastic nodule) although VMI at low-energy images showed lower overall image quality score, making the entire image evaluation difficult.

Recently, VMI is becoming to be applied to HCC evaluation. Peijie et al reported that the detectability for HCC with VMI at 40 to 70 keV images on HAP and PVP were well at low-energy images.^[26]^ Hanson et al^[27]^ reported that VMI at low-energy image improved liver lesion conspicuity than the low-tube voltage image, and VMI at the low-energy image was inferior to the low-tube voltage image in image quality and imaging noise. Although, these studies did not examine the HCC conspicuity between VMI at low-energy and low-tube voltage images. More recently, some studies have evaluated the detectability of HCC using VMI at several energy levels; they didn’t compare VMI with low-tube voltage CT.^[28,29]^ Our study is the first to investigate the early HCC enhancement detectability between VMI at low-energy and low-tube voltage images, and we showed that the low keV images increase contrast enhancement on HAP. VMI is reported to improve the detection of hypoattenuating tumors or washout of arterially hyper-enhancing tumors on PVP and/or DP^[30,31]^; the detection of HCC might be enhanced by combining low KeV VMI of PVP and DP.

Our study had some limitations. First, the majority of patients showed no pathological evidence. Although, we confirmed early enhancement on HAP, washout on DP, and hepatocyte phase hypointensity with EOB-MRI based on LI-RADS.^[4–6]^ The concordance rate between images and pathology based on LI-RADS is high, and imaging findings were possibly evidence for HCC even in the absence of a pathology diagnosis.^[32]^ Second, the body weight of patients in the present study (range: 34–68 kg; mean: 59 kg) was light compared with that of western people. Third, a direct comparison between VMI and EOB-MRI was not performed because EOB-MRI was used as one of the gold standards of imaging in the present study. Forth, the radiation dose for the 80 kVp and 140 kVp data was lower compared to the mixed 120 kVp data and VMI data, making it difficult to make fair comparisons among the evaluated image series. To ensure accurate comparisons, 80 kVp images need to be acquired at the same radiation dose as in this study using single-energy mode. Fifth, though this study used a single vendor, the image quality of VMI can vary significantly among different DE-CT systems. Greffier et al^[33]^ demonstrated that detector-based DE-CT systems have lower image noise and improved lesion detectability for low keV VMI compared to tube-based DE-CT systems. Additionally, deep-learning image reconstruction, which has recently been introduced, has shown promise in reducing image noise and improving the detectability of low keV VMI for fast-kV switching CT.^[34]^ Future research in these areas is desirable. Lastly, we were not able to assess specificity or accuracy because there were no hepatic lesions other than HCC in the present study.

## 5. Conclusion

In summary, VMI at 40 and 50 keV with DE-CT have significantly greater sensitivity for detecting small HCC hypervascularization compared with 80 kVp and blended 120 kVp based on the significant improvement of CNR of the images, although a little image deterioration was observed during HAP imaging.

## Acknowledgments

The authors thank Dr Mari Tokunaga, Dr Aya Takase, and Dr Yuki Komatsu for their assistance with image preparation. Additionally, the authors express immense gratitude to all colleagues who are radiology technologists represented by Hisashi Takeda in Isesaki Municipal Hospital for their helpful cooperation.

## Author contributions

**Conceptualization:** Tomoaki Ichikawa.

**Data curation:** Tomoaki Ichikawa, Keisuke Izuka, Takashi Ueno.

**Formal analysis:** Hiroyuki Morisaka.

**Methodology:** Tomoaki Ichikawa.

**Writing – original draft:** Haruomi Yamaguchi.

**Writing – review & editing:** Tomoaki Ichikawa, Hiroyuki Akai, Osamu Abe, Yoshito Tsushima.
